# The ‘Jekyll and Hyde’ of Gluconeogenesis: Early Life Adversity, Later Life Stress, and Metabolic Disturbances

**DOI:** 10.3390/ijms22073344

**Published:** 2021-03-25

**Authors:** Snehaa V. Seal, Jonathan D. Turner

**Affiliations:** 1 Immune Endocrine and Epigenetics Research Group, Department of Infection and Immunity, Luxembourg Institute of Health (LIH), L-4354 Esch-sur-Alzette, Luxembourg; snehaa.seal@lih.lu; 2 Faculty of Science, Technology and Medicine, University of Luxembourg, L-4365 Esch-sur-Alzette, Luxembourg

**Keywords:** glucose, glycogen, gluconeogenesis, early life adversity, acute stress, chronic stress, psychosocial stress, hypothalamus-pituitary-adrenal axis, ageing, immuno-senescence, inflamm-ageing, developmental origins of health and disease

## Abstract

The physiological response to a psychological stressor broadly impacts energy metabolism. Inversely, changes in energy availability affect the physiological response to the stressor in terms of hypothalamus, pituitary adrenal axis (HPA), and sympathetic nervous system activation. Glucocorticoids, the endpoint of the HPA axis, are critical checkpoints in endocrine control of energy homeostasis and have been linked to metabolic diseases including obesity, insulin resistance, and type 2 diabetes. Glucocorticoids, through the glucocorticoid receptor, activate transcription of genes associated with glucose and lipid regulatory pathways and thereby control both physiological and pathophysiological systemic energy homeostasis. Here, we summarize the current knowledge of glucocorticoid functions in energy metabolism and systemic metabolic dysfunction, particularly focusing on glucose and lipid metabolism. There are elements in the external environment that induce lifelong changes in the HPA axis stress response and glucocorticoid levels, and the most prominent are early life adversity, or exposure to traumatic stress. We hypothesise that when the HPA axis is so disturbed after early life adversity, it will fundamentally alter hepatic gluconeogenesis, inducing hyperglycaemia, and hence crystalise the significant lifelong risk of developing either the metabolic syndrome, or type 2 diabetes. This gives a “Jekyll and Hyde” role to gluconeogenesis, providing the necessary energy in situations of acute stress, but driving towards pathophysiological consequences when the HPA axis has been altered.

## 1. Introduction

The psychophysiological stress reaction is the manner in which the body reacts to an external stressor that requires a fight or flight response, disturbing physiological homeostasis. The stress reaction is primarily mediated by catecholamines and glucocorticoids. Initially, they maintain homeostasis and contribute to our overall survival. However, over the long-term, increased exposure to stress (allostatic load) has negative consequences [1]. Activation of stress reaction mobilises stored energy, induces immune cell trafficking, and biases the immune response as well as increasing heart rate and blood pressure, ensuring that oxygen and energy sources are available where needed.

Carbohydrate metabolism, in particular glucose homeostasis, is a key component of the metabolic reaction to an external stressor. Stress hormones such as the glucocorticoids play an important role in maintaining glucose homeostasis mediated by hepatocytes [2,3], where glycogenesis (storage of glucose in glycogen chains), glycogenolysis (glucose release from glycogen), gluconeogenesis (de novo glucose production), and glycolysis (ATP release as glucose is converted to pyruvate and ATP) are balanced to maintain plasma glucose levels with tightly controlled parameters [4,5]. Under normal physiological conditions, insulin, the only known glucose-lowering hormone, is principally counterbalanced by glucagon to control glucose homeostasis [5]. Plasma insulin, glucagon, and epinephrine levels are all intimately linked to blood glucose levels [6]. To maintain plasma glucose, insulin activates glucose consuming processes (glycolysis, glycogenesis) while glucagon and adrenaline increase glucose production (gluconeogenesis, glycogenolysis). During fasting, gluconeogenesis is triggered by glucagon via the cAMP/PKA/CREB/CRTC2 signalling pathway. This terminates at the peroxisome proliferator-activated receptor γ coactivator 1 α (PGC1α), which in turn coactivates transcription factors, including hepatocyte nuclear factor 4 α (HNF4α), forkhead box O1 (FOXO1), and GC receptor (GR), to activate hepatic gluconeogenesis [7]. When blood glucose levels rise after a meal, the rise in insulin levels inhibits gluconeogenesis by down-regulating the transcriptional mechanisms (FOXO1, PGC1α, and CRTC2) [7] as well as by activating glucose uptake by peripheral tissues.

The metabolic syndrome (MetS) is the umbrella term that includes impaired glucose metabolism, obesity, and hypertension, all of which increase the risk of type 2 diabetes (T2D) [8]. Over the last decade, MetS has been linked to chronic diseases by altered metabolic and pro-inflammatory pathways, and it has been demonstrated that it originates in early life, with early life socioeconomic position being the strongest lifelong driver of MetS [9]. Early life adversity (ELA) is a broad term that covers all negative experiences affecting an infant’s security or safety and inducing a large stress response. It ranges from growing up in a dysfunctional household, abuse or maltreatment to victimisation, bullying, or exposure to crime [10,11] and low socioeconomic status. Parental BMI, acting through a shared cultural environment and learned family eating patterns, influences adiposity, BMI, and lipid profiles in their children [9,12], passing through maternal education [13]. There is a strong epidemiological link between early life stress or adversity and T2D [14,15], as well as hypertension and dyslipidaemia [16], that was clear in meta-analyses over the last half-decade [17,18]. It is probable that the risk of long-term metabolic disturbances passes through “programming” during sensitive developmental windows during early life. Such critical windows of developmental plasticity permit the body to adapt to the environment in which it is developing, and are thought to be mediated by epigenetic changes, potentially in systems such as the HPA axis that are sensitive to the external environment [19].

Here, we review how the early life period programs the HPA axis, and how it interacts with metabolic pathways at baseline and under acute and chronic stress. We suggest that the link between exposure to chronic stress in early life and changes in the metabolic profile later in life with an increased risk of metabolic syndrome and type 2 diabetes may occur either through changes in gluconeogenesis in the liver, or through the manner in which HPA axis glucocorticoids regulate gluconeogenesis. “Classic” results have been generated over previous decades, however, only recently has detailed molecular evidence started to become available as to how glucocorticoids regulate gluconeogenesis [7], and to date, their exact role in the development of MetS after exposure to ELA has been rather under-explored.

## 2. The Physiological Response to External Psychosocial Stressors

### 2.1. The ANS and the HPA Axis Stress Response

Exposure to a stressor activates the paraventricular nucleus of the hypothalamus to secrete corticotropin-releasing hormone (CRH), which in turn activates two synergistic stress response systems, the rapidly responding autonomic nervous system (ANS) [20,21] with an associated catecholamine release and the second, slower arm of the stress response is the hypothalamus–pituitary–adrenal (HPA) axis [22]. The catecholamines adrenaline and noradrenaline act rapidly and transiently upon stress exposure by increasing the heart rate and raising blood pressure. The sympathetic branch (SNS) of the ANS directly activates preganglionic neurons that project from CRH-containing neurons in the PVN, through noradrenergic centres in the locus coeruleus in the brainstem, then projecting directly through sympathetic preganglionic neurons in the adrenal medulla chromaffin cells from where catecholamines are secreted [23]. This is tempered, controlled, and negated by the parasympathetic branch (PNS) of the SNS, again projecting from the locus coeruleus, returning the system to homeostasis. The locus coeruleus integrates signals and balances SNS and PNS activity through activation of the α1- and α2-adrenoceptors on the sympathetic and parasympathetic neurons, respectively [24,25]. Furthermore, SNS activation provides positive feedback, further increasing CRF secretion from the PVN [26]. The hippocampus, now thought to be a key element of the approach-avoidance system which involves weighing probabilistic profits and losses for an experience, thereby playing a prime role in anxiety generation. Hence, it is also part of the conscious stress response [27] and feeds direct inhibitory signals into the PVN, influencing basal GC levels, circadian GC rhythms, as well as inhibiting the HPA axis stress response [28,29].

The end product of the HPA axis are the glucocorticoids (GC), principally cortisol in humans and corticosterone in rodents, that act through their cognate receptor, the glucocorticoid receptor (GR). The presence of 17-α-hydroxylase (CYP17) in the human adrenocortical zona fasciculata means that in addition to cortisol, humans secrete a small amount of corticosterone. The physiological function of corticosterone in man remains to be fully elucidated despite it having analogous metabolism. The two molecules are differentially shuttled across membranes in humans by ATP binding cassettes (ABC), resulting in differential responses to them in a tissue specific manner [30]. These steroid glucocorticoids subsequently regulate, e.g., inflammation, lymphocyte trafficking, metabolic, cardiovascular, and behavioural processes, amongst others [31], and are regulated by a hormonal cascade which is initiated by the hypothalamic nuclei. Circadian messages from the suprachiasmatic nucleus (SCN) are integrated with physical, emotional, and cognitive reactions in the PVN [32,33]. Activated neurons in the PVN secrete CRH. The cascade is propagated via adreno-corticotropic hormone (ACTH) released from the anterior pituitary gland, which in turn stimulates the adrenal cortex to release glucocorticoids [34,35].

**Figure 1:**
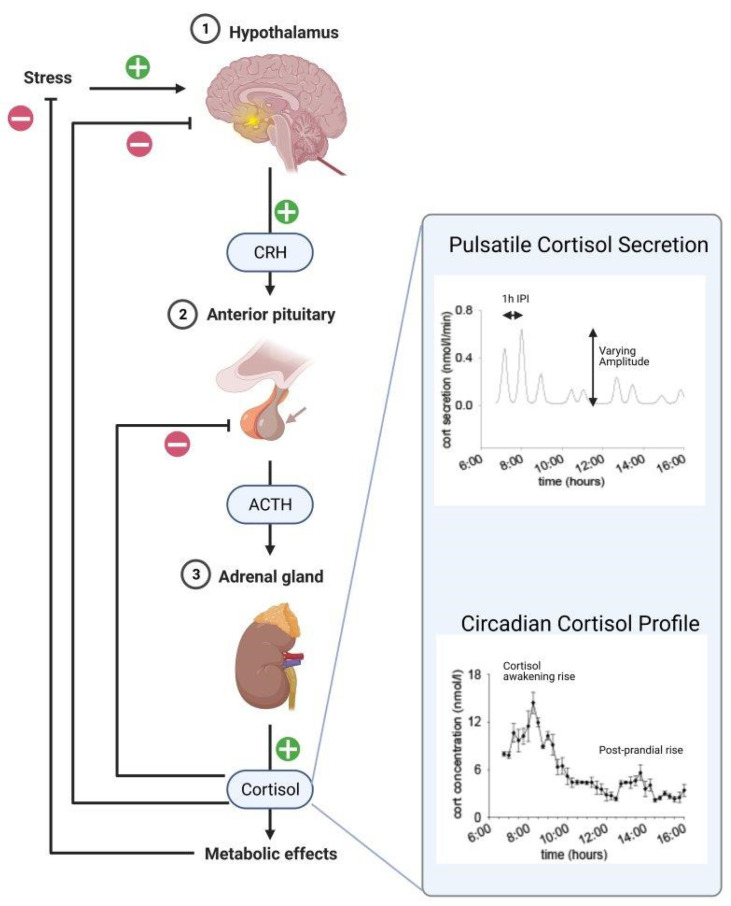
The hypothalamus–pituitary–adrenal (HPA) axis controls both ultradian and circadian cortisol rhythms to generate metabolic effects that help the body combat stress and re-establish homeostasis post stress. Pulsatile cortisol profiles adapted from [37].

Cortisol and corticosterone have ultradian and circadian rhythms which are chiefly regulated by the HPA axis (Figure 1). Circadian cortisol and corticosterone concentrations peak around waking; cortisol peaks in the early morning hours, while corticosterone levels peak mid-afternoon in nocturnal rodents. This circadian rhythm overlies an ultradian rhythm of the complete HPA axis signalling cascade. The frequency of secretory episodes is relatively stable at one every ~1 h while the amplitude of the secretory episode, and hence the mass secreted, provides variation in the measured concentrations [36,37,38], as shown in Figure 1. The corollary to the pulsatility model is that rapidly rising glucocorticoid levels in the secretory phase induce the necessary glucocorticoid receptor-mediated negative feedback that terminates the hormonal pulse, then, after a constant time interval (the inter-pulse interval), the SCN and the PVN trigger the subsequent pulse [39]. Thus, the HPA axis in conjunction with the ANS provides the molecular weapons needed to elicit a fight/flight reaction in response to stress.

In early life, the stress hypo-responsive period (SHRP) is a particular period when the HPA axis is poorly responsive. This is between postnatal-days 3–14 (*R. norvegicus)*, and 1–12 (*M. musculus*) [40]. This period is characterised by extremely low baseline plasma corticosterone levels, as well as a reduced ACTH and corticosterone response to stress. This period is complemented by a second critical period. During adolescence, gonadal hormones exert an organizational influence on the HPA axis [41]. As outlined in Section 3 stress in these periods, particularly the SHRP, leads to behavioural and neuroendocrine abnormalities, as well as depression and anxiety disorders in a sex-dependent manner.

### 2.2. Metabolic Adaption

**Figure 2:**
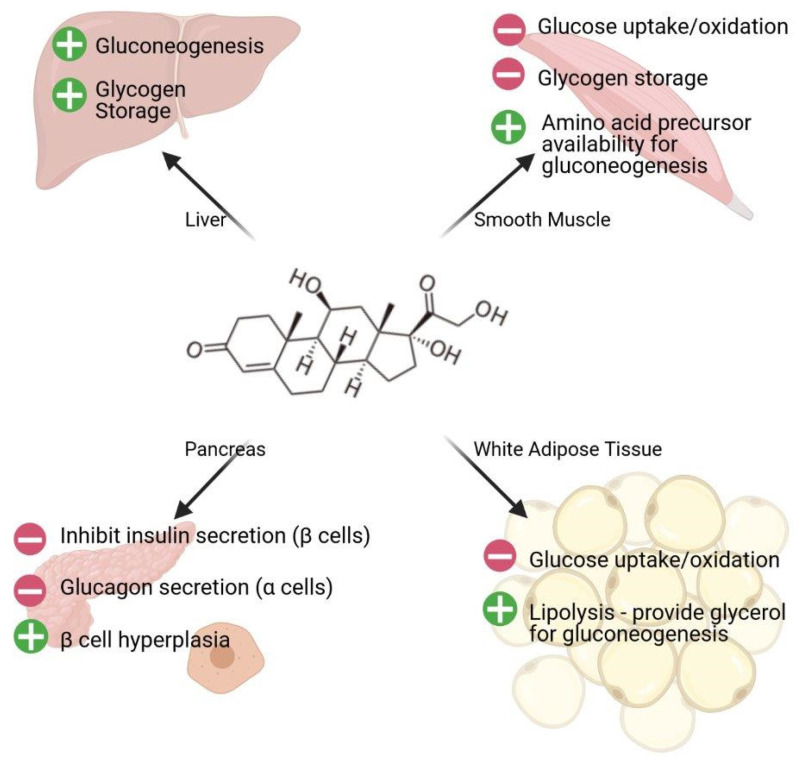
Cortisol triggers a cascade of events that affect glucose homeostasis. The liver, skeletal muscles, white adipose tissues, and pancreas play a key role in ensuring continuous supply of useable energy for the fight/flight response.

Metabolic adaptation during stress aims to preserve glucose, providing a short, transient increase in blood glucose levels, temporarily enhancing cognitive processes [42]. In addition to gluconeogenesis, glucose uptake is reduced in white adipose tissues and skeletal muscles under stress conditions (reviewed in [42] (Figure 2)).

Secondary active transport and facilitated diffusion are the two main ways in which glucose is trafficked within the body. Secondary active transport makes use of ATP to distribute glucose in the kidneys. Facilitated diffusion relies on special membrane proteins that allow the ferrying of glucose molecules against a concentration gradient, which may/may not be insulin driven.

GLUT receptors predominantly govern the uptake of glucose into the cells. There are 14 distinct glucose transporters, of which only GLUT 1–4 are particularly interesting [43]. GLUT 1, a key receptor in the brain and GLUT 3 have been reported to have high preference for glucose. GLUT 2 and GLUT 4 dictate the uptake of glucose in the pancreatic cells and muscles, respectively [43,44].

Absorbed glucose is directed to enter the glycolytic cycle, the final product of which is pyruvate. The synthesised pyruvate is converted into either lactate or carbon dioxide and water depending on the absence or presence of oxygen, respectively. Normal physiological conditions strike a balance between the lactate and pyruvate concentrations. Chronic stress increases the net lactate and pyruvate concentrations [45]. This happens so that the produced pyruvate can either enter the glycolytic cycle to produce glucose or be metabolised to generate high concentrations of lactate due to reduced action of pyruvate dehydrogenase (PDH), which normally functions under anaerobic conditions [46]. Chronic stress attenuates the activity of PDH owing to phosphorylation by increasing concentrations of PDH kinase [47]. Stress also causes lipolysis, which provides gluconeogenic substrates [48]. Allostatic adaptations during stress also result in increased activity of the citric acid cycle due to readily available acetyl coenzyme-A produced by the oxidation of fatty acids. This increased activity of the citric acid cycle also supplies substrates for gluconeogenesis such as oxaloacetate. Thus, these interconnected metabolic pathways adapt during stress to ensure that the body, in particular the brain, has a useable glucose supply.

### 2.3. Metabolism and Acute Stress Interactions

Acute laboratory stressors such as the Trier Social Stress test (TSST) activate the HPA axis, inducing cortisol secretion and then the negative feedback loop, bringing cortisol levels back to baseline [49]. When the TSST was first developed, the role of energy availability was rapidly investigated. It was initially reported that an 8 h fasting period prior to a TSST significantly reduced HPA axis reactivity, while glucose supplementation 1h before the paradigm significantly increased cortisol and HPA axis reactivity [50]. Furthermore, when this was extended to food components such as protein or fat, the fasting-induced blunting of the HPA axis response was not restored due to immediate unavailability of useable energy. Moreover, there was a strong link between the increase in cortisol levels during the stress paradigm, and the increase in blood glucose after administration of the glucose bolus [51]. When the mechanisms were further dissected in rats, hypothalamic nuclei such as the ventromedial and paraventricular nuclei (VMN, PVN, respectively) stood out. It was proposed that the high insulin and glucose levels observed after glucose loading stimulated VMN, inputting into the PVN, subsequently activating the HPA axis. Importantly, this was pharmacologically validated by colchicine (VMN activity antagonist) disrupting the fasting-induced reduction in HPA axis responsiveness [6,52] and moreover, low blood glucose levels significantly inhibit VMN and PVN activity, consequently attenuating HPA axis activity and reactivity. The differential response to glucose, fat, and protein loading prior to the TSST suggest that, similar to the rat, central mechanisms are also involved in the human situation rather than metabolic pathways such as the citric acid cycle [51].

At baseline, both hypo- and hyper-glycaemic states alter the HPA axis. In both the fasting and post-prandial state cortisol secretion was increased. Deconvoluting cortisol concentration to access the underlying HPA axis activity, hyper- and hypoglycaemic states had no effect on the number of secretory events, the inter-pulse interval, secretory burst length, or cortisol half-life, however, they modulated the mass secreted during each event [53,54]. There is, however, a gap in the literature on how a stressor such as the TSST activates gluconeogenesis. On the other hand, blood glucose levels are also dependent on catecholamine levels. Release of epinephrine induces a rapid rise in blood glucose levels through a combination of reduced glucose uptake in insulin-dependent tissues as well as a temporary increase in hepatic glucose production from both gluconeogenesis and glycogenolysis. Glycogenolysis wanes rapidly, while gluconeogenesis is mainly responsible for the resultant hyperglycaemia [55]. Therefore, it can be postulated that the status of the HPA axis may be extracted from blood glucose records, and vice versa, and that the nervous system plays a significant role in maintaining homeostasis/allostasis.

### 2.4. Metabolism and Chronic Stress

Synthesis of glucose using “non-carbohydrate sources” occurs by gluconeogenesis, a central metabolic pathway that also operates during starvation. Stress regulated glucocorticoids transcriptionally trigger key players of the gluconeogenic pathway such as glucose-6-phosphatase (G6Pase), pyruvate carboxylase, pyruvate carboxy kinase (PEPCK), and fructose 1,6 bisphosphatase (FBPase). Interestingly, it has been shown that a doubling in PEPCK expression can lead to insulin resistance, and a seven times increase can result in hyperglycaemia [56]. The GC-GR transcriptional dependence is clearly demonstrated in mice lacking hepatic GR that show an exaggerated hypoglycaemia after fasting as neither PEPCK, pyruvate carboxylase, nor G6Pase can be upregulated [57], although all three genes are involved in gluconeogenesis, G6Pase is, however, also involved in hepatic glucose release from glycogenolysis [58]. This occurs through direct GR interactions with genomic glucocorticoids response elements (GREs). GR ligation and DNA binding provides a “transcriptional hub” where coactivators such as GR-associated PGC-1α, FOXO1, and HNF-4 interact to provide the full gluconeogenic response and expression of G6Pase and PEPCK (reviewed in [3]).

The concentration of other gluconeogenic substrates like oxaloacetate and pyruvate also increase during stress owing to increased activity of hormones such as glutamic oxaloacetatic transaminase (GOT) and glutamic pyruvic transaminase (GPT). Thus, chronic stress increases glycogenolysis and gluconeogenesis, while it downweighs glycogenesis and insulin sensitivity, resulting in a diabetogenic physiological state.

### 2.5. Glucocorticoids and Diabetes

Type 2 diabetes is largely the result of uncontrolled hepatic gluconeogenesis [59]. When the glucocorticoid actions are impaired by mutations in the GR, mice are protected from experimental models of hyperglycaemia and metabolic syndrome, and have reduced gluconeogenic gene expression. While the implication of glucocorticoids in T2D is well established, the underlying mechanisms are still not known, although there are data that suggest changes in expression of the GR itself [60], cofactors such as PGC-1 alpha [61], and inactivation of cortisol by 11-beta HSD, reducing GC signalling [62,63]. GC effects are also seen in the pancreas.

During metabolic dysfunction, insulin secreting pancreatic beta cells can no longer compensate for hyperglycaemia, and long-term exposure to high levels of exogenous or endogenous GC during therapeutic administration of Cushing’s syndrome, respectively, reduce insulin secretion and induce a diabetes-like phenotype [64]. Overexpression of the GR in pancreatic beta cells leads to reduced insulin secretion associated with an impaired glucose tolerance and eventually hyperglycaemia [65]. Moreover, GC signalling through the GR is a trigger of insulin resistance in muscles, supressing GLUT 4 translocation to the cell surface, as well as downregulating glycogen synthesis [66]. It would appear that the GR interacts directly with both the insulin and insulin-like growth factor 1 (IGF-1) pathways [67], although detailed mechanistic studies of GC effects in muscle carbohydrate metabolism are severely lacking [3].

The interaction between the HPA axis and T2D is bidirectional. There is a clear link between T2D, diabetic complications, and HPA axis hyperactivity [68]. Furthermore, hyperglycaemia from T2D can dysregulate the HPA axis, subsequently increasing the risk of major depression [69]. Mechanistically, this passes through altered secretion of ACTH from the pituitary gland, leading to altered GC levels [68,69]. In a series of particularly well designed experiments, Mosili et al. demonstrated that as rats entered a prolonged pre-diabetic state baseline ACTH levels under non-stressful conditions dropped, but GC levels were significantly elevated [70]. The HPA axis negative feedback loop should, under normal conditions, bring down GC levels when ACTH levels are low [71] suggesting that the negative feedback is somehow impaired [68]. Furthermore, when Mosili et al. induced a chronic stress together with pre-diabetes, the rats were unable to mount a normal ACTH or GC response to subsequent acute stressor, further consolidating their observation of either impaired GC signalling or negative feedback [70]. Interestingly, diet would appear to affect the adrenal gland as increased GC secretion, adrenal cortical hyperplasia, and increased steroidogenesis have been reported in T2D- and obesity-inducing high fat diets [72]. Furthermore, certain sugars such as fructose can bind the glucose transporter and can pass the blood–brain barrier and be absorbed in both the hippocampus and hypothalamus, activating the HPA axis [73,74]. Thus, poorly controlled T2D can result in an impaired HPA axis, which in turn creates a vicious cycle, where excessive GCs antagonise glucose homeostasis and insulin sensitivity.

## 3. Early Life Adversity

### 3.1. Early Life Adversity and Changes in the HPA Axis

In all societies studied so far, ELA is prevalent, with, for example 59% of the US population reporting at least one adverse event in the BRFSS (Behavioural Risk Factor Surveillance System) study [75]. ELA has broad long-term consequences on the neuroendocrine, immune, and metabolic systems (reviewed in [11]), as well as on neuroplasticity and neuronal morphology altering the overall cerebral maturation trajectory (reviewed in [76]). Although the literature is somewhat unclear and contradictory, the most useful classification proposed to date [76] has divided early life stressors into broad categories. “Mild” stress in the neo- and pre-natal period appears to induce HPA-axis hyperactivity, such as increasing the cortisol responses to a standardized stressor in pre-adolescent children [77,78]. Slightly higher “moderate” stress levels linked to more clear forms of early life adversity (e.g., frequent emotional maternal withdrawal, corporal punishment, or interparental aggression) increasing both the baseline cortisol level [79], and the HPA axis response to stress [80]. Severe early life stress or adversity, e.g., institutionalization, neglect, abuse, or deprivation, lowered basal cortisol levels [81,82] and blunted HPA axis reactivity [83,84]. Hypocortisolism and reduced HPA axis responsivity was initially proposed to be either due to a reduced pituitary response to hypothalamic CRF [85] or by the hypersensitivity of the final glucocorticoid target tissues and the HPA axis tissues in the negative feedback loop [86]. The latter would appear to be excluded, since in our EpiPath institutionalization/adoption “severe” early life stress cohort, peripheral glucocorticoid receptor signalling and functionality was essentially preserved and indistinguishable from non-exposed controls [87]. It should, however, be remembered that the literature has not come to a definitive conclusion as to the exact effects or classification of the different forms of ELA.

**Figure 3:**
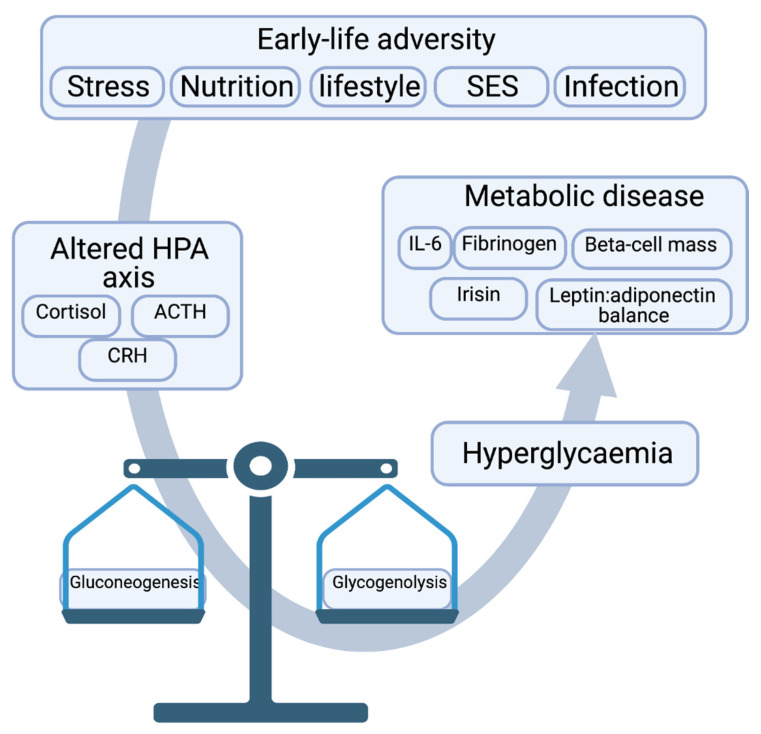
Early life adversity dysregulates the HPA axis and its key effector molecules, which in turn disrupts glucose homeostatic balance, leading to hyperglycaemia and metabolic syndrome if left unchecked.

The situation may be somewhat more complicated and dependent on the timing of the adversity. Adversity in early childhood was associated with a decreased hippocampal volume, whilst prefrontal cortex volume was reduced if exposed during adolescence [88,89]. Psychopathologically, exposure to adversity or trauma before age 12 increased the lifelong risk of developing major depressive disorder, but when occurring between age 12 to 18, the risk of PTSD was increased [90]. It has been suggested that since the human hippocampus is not fully developed before age 2, the frontal cortex primarily matures between age 8–14 and the amygdala continues developing until early adulthood [91], and the hippocampus is most probably the brain area most affected by early life stress [76]. The sensitivity of the hippocampus to ELA is particularly important, as, outlined above, it plays a key inhibitory role in PVN activation of the HPA axis. Hence, ELA renders the HPA axis impaired, which in turn causes insidious changes to the stress response mechanism along with glucose metabolism, eventually contributing to MetS (Figure 3).

There is more to the HPA axis than reactivity to a laboratory stressor. ELA was initially reported to be associated with elements of the cortisol diurnal rhythm such as the cortisol awakening rise (CAR) [92], however, the most recent meta-analysis suggests that this is not the case [93], although the meta-analysis of the stress-induced changes was much stronger [94]. The weakness in the CAR meta-analysis was probably due to large heterogeneity between the techniques employed between the different studies.

### 3.2. Early Life Adversity, Diabetes and the Metabolic Syndrome

MetS has been shown to predict not only cardiovascular mortality, but also the progression to full type 2 diabetes (T2D) [95]. There is growing evidence that early life nutritional and psychosocial stress or disadvantage determine the trajectory and transition into metabolic dysfunction, MetS, and T2D later in life [9]. There are two principal components of the early life socioeconomic position that contribute to lifelong MetS and diabetic risk—Early life nutrition and early life stress. Reports from ourselves and others of early life adversity have shown effects on either the metabolic profile, obesity, or type 2 diabetes [96,97,98]. Socioeconomic status in early life has a similar effect [15], and is associated with T2D 50 years later [99]. ELA also predisposes individuals towards a chronic inflammatory phenotype [98,100,101,102]. Furthermore, cardiometabolic disease markers such as fibrinogen, C-reactive protein, and interleukin-6 were elevated [17,103], as were endothelial dysfunction markers such as ICAM-1, E-selectin [104], as well as clinical measures such as arterial stiffness [105] or poor blood pressure trajectories with age [106]. Recently, this was replicated by Chandan et al. (2020), confirming the role of ELA in determining “a significant proportion of the cardiometabolic and diabetic disease burden may be attributable to maltreatment” [107,108].

Although there are few mechanistic data available, these metabolic abnormalities may be due to changes in circulating adipokine levels. ELA has been directly associated with an increased leptin:adiponectin ratio [109]. Leptin, secreted by adipocytes, regulates energy balance through decreasing appetite and is associated with the metabolic syndrome [110,111], whilst adiponectin, has insulin-sensitizing effects. Low adiponectin levels are associated with type 2 diabetes and insulin resistance [111,112]. Furthermore, Irisin levels were increased by ELA [109]. Irisin, mediates glucose metabolism as well as exercise-related energy expenditure and is a peroxisome proliferator-activated receptor-γ coactivator 1-α (PGC-1α)-dependent myokine [109,113]. Glucocorticoids may also play a role, as, in utero exposure to maternal under nutritional affects beta-cell number and function lifelong in a manner dependent upon GR and GC since deletion of the GR in foetal pancreatic cell abrogated this effect [3]. These changes may, in part be due to the effects of ELA on the methylation of genes involved in obesity and metabolic pathways [114,115]. Furthermore, adverse early life conditions induce lifelong changes in gene transcription [116]. Low SES, for example, has been associated with inflammatory and diabetic genes such as *TLR3* [117], *NLRP12* [118], *F8* [119], *KLRG1* [120], *CD1D* [121], as well as the stress-associated genes *OXTR* [122], *FKBP5* [123], and *AVP* [124], suggesting, as we have previously proposed, that a negative early life environment acts through inflammatory pathways that are also associated with T2D, targeting pathophysiological factors such as stress and inflammation, and participating in the aetiopathology of T2D [108]. Thus, it appears logical to conclude that ELA can effectively alter glucose homeostasis and that the aetiology of MetS and eventual T2D may have strong roots in ELA. Moreover, ELA would appear to be associated with more advanced or complicated diabetic-pathologies requiring more aggressive management [125].

### 3.3. Glucose Metabolism, Allostasis and Allostatic Load

Ever since Hans Selye described the “general adaptation syndrome” as our response to external stressors [126], there has been a paradox. The ANS and HPA axis protect in the short-term, but over the long-term they may accelerate disease as well as causing lasting damage. Allostatic Load (AL) is fundamentally a chain of causal events from the primary stress response of SNS and HPA axis activation with epinephrine and cortisol secretion, inflammation [127], leading to secondary markers of stress exposure including hyperglycaemia, hypertension, hyperlipidaemia, and central adiposity. The AL cascade, the overall sequence of responses, as well as their contribution to disease development, are not fully understood, and somewhat under-investigated, although markers of AL are associated with increased glycaemic measures in women [128]. Similarly, rat chronic stress models, a proxy for AL, consistently report high blood glucose levels up 6 months later [46]. In allostasis these rats also had dysregulated glucose metabolism pathways, in particular increased gluconeogenesis [46]. Furthermore, this stress induced hyperglycaemic state resulted in impaired glucose tolerance and reduced insulin sensitivity [46]. This may be due to metabolic memory, which causes the body to recall the influence of metabolic regulators for a much longer duration. This, in conjunction with persistent stress, can lead to maladapted allostasis and eventually AL. Prolonged AL can give rise to metabolic syndrome. There is also recent evidence that diet may act as a stressor. Increased glycaemic load (i.e., dietary sugar intake) is associated with increased markers of AL, particularly in women, suggesting that dietary carbohydrate intake may contribute towards dysregulation of the AL response [128]. It is well established that carbohydrate intake can stimulate the ANS [129], and hypothesised to cascade down increasing AL markers and blood glucose levels [128]. Thus, chronic stress/AL can adversely affect glucose metabolism and mount a faulty bodily adaptation that ultimately leads to MetS.

## 4. Gluconeogenesis at the Crossroads between Adversity and Metabolism

It is well established that energy metabolism and psychosocial stress are intimately intertwined. It would also appear that psychosocial adversity in early life sets the individual on a negative trajectory towards either MetS or T2D. The available literature suggests that ELA can effectively alter glucose homeostasis and participate in the aetiology of MetS and eventual T2D. It is possible that MetS and T2D may have very strong roots in the early life environment, with ELA as a strong driver of the eventual diabetic phenotype. This suggests that gluconeogenesis, originally named because of the intimate link between corticoid levels and glucose levels, may be at the heart of the mechanism. We suggest that this may be a double-edged sword. Gluconeogenesis is an integral part of energy homeostasis in response to an external stressor. However, it may show its true “Jekyll and Hyde” nature when the HPA axis is perturbed. Furthermore, the interaction between the HPA axis, glucocorticoids, and mechanisms of glucose homeostasis, such as gluconeogenesis or insulin resistance, may be the link between ELA and lifelong metabolic disturbances. Indeed, one consequence in the neonate of maternal separation is a rapid drop in blood glucose levels. This, together with increased ghrelin (“hunger hormone”) levels may actually trigger HPA axis activation, and glucose supplementation during maternal separation reverses the phenotype, confirming the link and providing a potential mechanism to counteract it [130].

Energy homeostasis during psychosocial stress is somewhat underexplored, however, a number of recent studies have started to investigate the full nature of the bi-directional regulation in more detail. There is a wealth of data on how glucose availability modulates the corticosteroid response to psychological and psychosocial stressors, however, the data is sparse or inexistent in the reverse direction. Glucose or levels of gluconeogenesis need to be determined after stress. Furthermore, they need to be recognised as genuine measures, e.g., the Trier Social Stress test, providing insight into how the stress axes interact with energy homeostasis. Elucidating these GC-glucose interactions during laboratory stressors is now essential. We need to initially understand the normal blood glucose response to an external stressor. This will subsequently permit investigation of glucose-cortisol coupling in situations such as exposure to ELA where the HPA axis has been significantly programmed, with lifelong changes in reactivity, setpoint, and secreted hormone levels. Recent work on the direct transcriptional control of gluconeogenesis [7], together with recent interest in stress-energy balance [131], open the field for more detailed investigation of the bi-directional regulation of these two essential physiological systems. Thus, it is extremely important to identify if the “Jekyll” or the “Hyde” of gluconeogenesis is at play, and how it balances energy homeostasis under stress, while avoiding gluconeogenesis driven T2D.

## 5. Conclusions

In light of the presented narrative, it is clear that there is a direct link between ELA, the HPA axis, glucose metabolism, MetS, and potentially T2D. It is now essential to understand how early life programming of the HPA axis, with lifelong changes in glucocorticoid secretory profiles, influences energy metabolism, and processes such as hepatic gluconeogenesis. To do this, and to provide the missing piece of the puzzle we need to consider glucose and energy homeostasis as genuine output measures in standardised laboratory psychological stress tests such as the TSST or the socially evaluated cold pressor test. We need to initially investigate the normal physiological interaction between the HPA axis (or the complete stress system), and energy homeostasis. We hypothesise that lifelong programming of the HPA axis after ELA will fundamentally alter hepatic gluconeogenesis, inducing hyperglycaemia, and the significant lifelong risk or MetS or T2D.

Early life developmental programming of the MetS or T2D risk dovetails nicely into the developmental origins of Health and Disease (DOHaD) model developed by David Barker. The DOHaD model has evolved over the last years into the current “three hit model” [132,133]. In the current concept the three “hits” are defined as (i) invariable genome that fixes genetic risk at conception, (ii) the early life environment during which many biological systems are modulated or adapted to the environment the individual is born into, and (iii) the later-life environment where a perturbation tips the balance between health and disease. The first and second hits produce a phenotype that remains latent or quiescent until the third hit crystallises the risk and initiates the health-disease transition. In our ELA-metabolic disease paradigm, we see the early life adversity during the SHRP as the second hit and the cluster of metabolic abnormalities as the final disease phenotype. Indeed, there is now evidence that ELA leaves a faint metabolic imprint almost immediately, although the full syndrome does not appear until much later in life, but usually becomes manifest no earlier than at adult age [134]. This mirrors the observation from pre-term infants, where increased baseline HPA axis activity, reduced stress reactivity, and components of the metabolic syndrome are seen in later life [135]. This may, however, be linked to the use of synthetic glucocorticoids such as dexamethasone in the perinatal period. However, Vargas et al. demonstrated that early life stress could concurrently increase HPA axis activity, and mild metabolic alterations, that were significantly increased with a subsequent environmental challenge akin to the third hit, although their model suggested that it was independent of GC [136].

One potential limitation to our postulate is that chronic stress has been shown to bias feeding choices in both animals and humans to addictive foods such as high sugar and high fat content foods, which can directly contribute to MetS over the years (reviewed in [137]). This could be mediated by alterations in the glucose metabolism, HPA axis related changes, etc. This limitation can be further dissected by studying laboratory models of chronic stress that are only fed standard chow diet to see if gluconeogenesis is indeed altered. It is also interesting to note that chronic stress is frequently accompanied by comorbidities like depression [138], which decrease sucrose preference in depressive animals and is often used as an index of anhedonia [139], providing a conflicting perspective with respect to chronic stress and diet.

Thus, we suggest a “Jekyll and Hyde” role to gluconeogenesis, providing the necessary energy in situations of acute stress, but driving towards pathophysiological consequences when the HPA axis has been altered. Furthermore, if our hypothesised link is correct, does this output have the predictive power to identify individuals at a higher risk of developing MetS at a later stage in life, before symptom onset?

## Data Availability

Not applicable.
